# WeavePop: a bioinformatics workflow to explore and analyze genomic variants of eukaryotic populations

**DOI:** 10.1093/g3journal/jkag039

**Published:** 2026-02-13

**Authors:** Claudia Zirión-Martínez, Paul M Magwene

**Affiliations:** Department of Biology, Duke University, PO Box 90338, Durham, NC 27708, United States; Department of Biology, Duke University, PO Box 90338, Durham, NC 27708, United States

**Keywords:** copy number variants, small variants, population genomics, eukaryotic, Snakemake, shiny, database, workflow, fungi, Python

## Abstract

Analyzing genomic variants in large datasets composed of short-read sequencing data is a process that requires multiple steps and computational tools, which makes it a complicated task that is difficult to reproduce across projects and laboratories. To address this need, we developed a reproducible and scalable Snakemake workflow called WeavePop, which aligns samples to selected references; obtains reference-based assemblies, annotations, and sequences; and identifies small variants and copy number variants in eukaryotic haploid organisms. All the results are integrated into a database that can be easily shared and explored through a graphical web interface provided alongside the workflow, making the discovery of variants in a population of study very simple. WeavePop is available from GitHub (https://github.com/magwenelab/WeavePop) for Linux operating systems. Here, we exemplify the use of WeavePop in a large collection of isolates of the pathogenic fungus *Cryptococcus neoformans*.

## Graphical Abstract

**Graphical Abstract:**
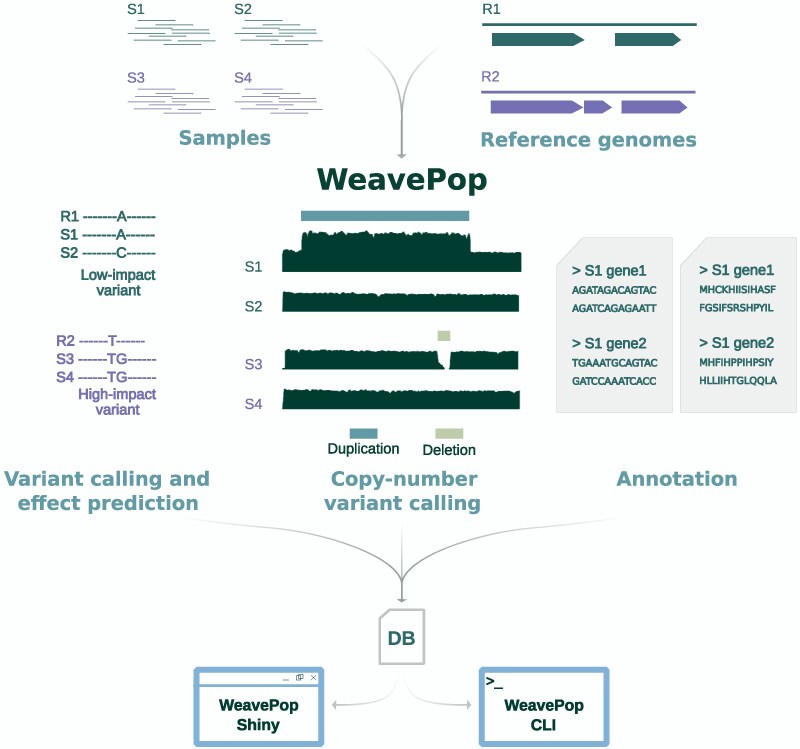

## Introduction

Sequencing whole genomes, particularly those of microbial organisms, has become a routine and relatively inexpensive procedure for many investigators and research teams. It is not unusual for a single research group to sequence tens to hundreds of genomes annually for various purposes, including population genomics, experimental evolution, mutational screens, and phylogenomics. In addition, for many model organisms, hundreds or thousands of whole-genome sequencing runs are available in public databases such as the National Library of Medicine Sequence Read Archive and the European Nucleotide Archive. Despite the ubiquity of genome sequence data, the computational process required to go from raw sequence reads to mapped genomes while also detecting both small-scale and structural variations remains a significant challenge due to the number of steps it involves and the complexity of the tools required. Moreover, ensuring that this process is reproducible and the resulting data is accessible to users with varying levels of computational expertise adds greater complications to the task.

To address this computational hurdle, we developed WeavePop (**W**orkflow to **E**xplore and **A**nalyze **V**ariants of **E**ukaryotic **Pop**ulations), a bioinformatics pipeline that carries out an extensive set of analyses to provide complementary information about genomic variation within a collection of eukaryotic haploid organisms. WeavePop detects and catalogs large-scale and small-scale variations by including reference-based read mapping (allowing for multiple references), variant calling, variant effect prediction, identification of repetitive sequences, and copy number variant (CNV) detection. To accomplish these tasks, WeavePop takes advantage of multiple field-standard tools in combination with new algorithms, bringing them together in a pipeline that facilitates their application to multiple use cases. This saves time and generates a diverse set of outputs that are convenient to evaluate directly or that can be passed to downstream analyses.

WeavePop is implemented as a Snakemake (v9.8.1; Mölder et al. 2021) workflow. The use of Snakemake to implement this pipeline makes it readable, scalable, reproducible, and easy to configure and execute. WeavePop is available through GitHub for Linux operating systems, and all of the required software tools are readily installed using predefined Conda environments (Conda Contributors 2025) managed by Snakemake.

The numerous outputs generated by WeavePop across multiple samples are combined into a single DuckDB (v1.3.0; Raasveldt and Mühleisen 2019) SQL database file, which can be readily shared, archived, and used to explore the output in a user-friendly manner. This database can be accessed via standard SQL syntax or using DuckDB client APIs for popular languages such as Python and R. In addition, we provide a local Shiny (v0.10.2; Posit Software 2022) web application that facilitates querying the database for variants and their predicted effects, CNVs, annotation, and gene sequences. In sum, using the databases produced by WeavePop allows a single lab or small research community to produce easily shared resources that facilitate exploration and analysis of genomic variation.

Here we discuss the structure and implementation of WeavePop. We then illustrate the utility of WeavePop by applying it to study genome variation in a large collection (1,016 samples) of *Cryptococcus neoformans. C. neoformans* is a fungal pathogen of worldwide concern for human health (Brown et al. 2024), and we illustrate the application of WeavePop to detect and catalog population genomic copy number variation in this species.

## Materials and methods

### Overview

**Fig. 1.**
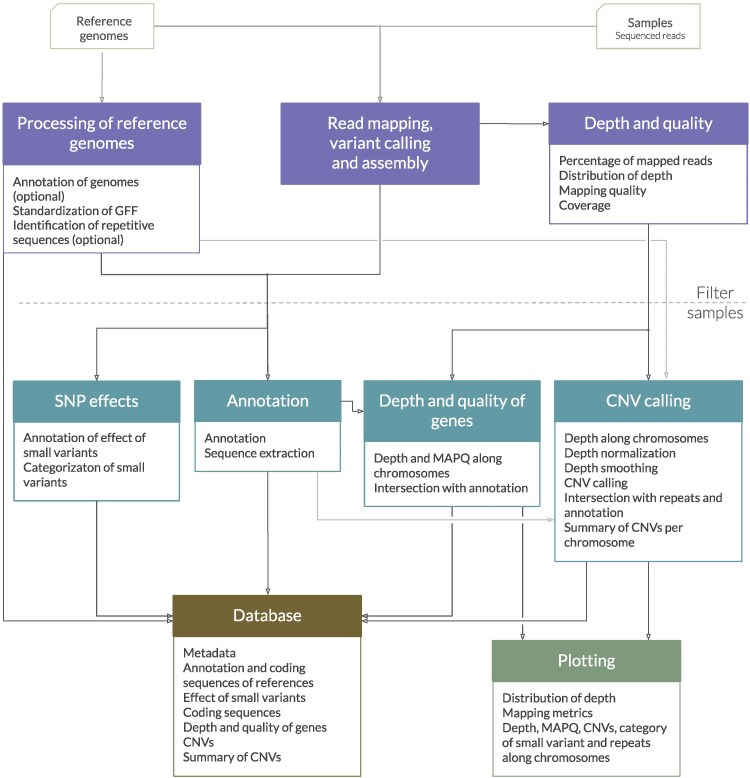
Overview of the modules in WeavePop. The base modules, in the top row, are for processing the reference genomes, read mapping, variant calling, and the analysis of the depth and quality. An optional step of filtering out low-quality samples can be included before the next steps. The optional modules, in the second row, are for the prediction of SNP effects, the annotation, the intersection of MAPQ and depth with annotated features, and CNV calling. The database module integrates the results of the previous modules into an SQL database. The plotting module generates graphs for each sample and summary graphs of the final dataset. The activation of a module automatically activates the required steps in the previous ones.

WeavePop aims to detect and catalog genomic variation between a set of samples and 1 or more suitable reference genomes. To exploit the availability of multiple reference genomes, WeavePop allows each sample to be aligned to a different reference genome, such as lineage-specific whole-genome assemblies, while at the same time facilitating comparisons across samples regardless of reference. For each sample, the workflow produces a read alignment, a reference-based assembly along with its annotation, the sequence of all genes, small variants (single and multiple nucleotide polymorphisms and indels) along with their predicted effects, and CNVs. An overview of the workflow is shown in Fig. 1, and the directed acyclic graph (DAG) of Snakemake tasks used to implement this workflow is shown in Supplementary Fig. 1.

### Snakemake description

WeavePop is implemented in Snakemake, a workflow management system written in Python, which organizes the execution of the steps required to generate target outputs. Snakemake facilitates both parallelization and efficiency. If the user wishes to re-execute the workflow with changes in the configuration and parameters, or if the workflow stops after a failure, Snakemake only repeats the execution of the steps whose results would be updated, saving time and storage. Snakemake also makes it possible to generate reproducible analyses because all the configurations are documented, and all the software tools used in multistep analysis are contained in Conda environments managed by Snakemake. After downloading WeavePop and installing Conda, the user only needs to run the following command to install Snakemake:

conda env create –file workflow/envs/snakemake.yaml

### Input

The pipeline requires each sample's paired-end short-read FASTQ files and a reference genome or genomes (see below the description of module 1). The relationship between each sample and the reference genome it should be aligned to is specified in a metadata table (Supplementary File 1).

A table that matches the chromosome sequence IDs of each reference genome with its conventional name is needed. This allows the comparison of chromosomes across reference genomes (Supplementary File 2).

The repetitive sequences of the reference genomes are identified using RepeatModeler (v2.0.1; Flynn et al. 2020) and RepeatMasker (v4.1.2; Smit 2021), which requires a database (FASTA file) of known repetitive sequences, such as the RepBase (Bao et al. 2015). The user can provide this file, allow WeavePop to use a mock database if an accurate identification is not needed, or skip this step.

Finally, 2 optional files can be provided: one to highlight the location of genes or genetic features of interest in the plots (Supplementary File 3) and a list of sample names to exclude from all analyses.

### Configuration

All the configuration options required to complete a run of WeavePop are specified in a single file (Supplementary File 4). The paths to the input files and output directory, as well as the parameters for the tools, are set in this file. The modular structure of the workflow permits the process to be run step by step. If no modules are activated, only the mandatory steps will be executed (the necessary steps of module 1 and modules 2 and 3). With these, one can evaluate the quality of the alignments and decide to adjust the filtering parameters (see module 3) before continuing. Modules 4 to 7 can be activated independently. To run every module of the analysis, only the database and the plotting modules need to be explicitly activated. Besides the modules mentioned above (analysis workflow), one can choose to integrate the results of multiple datasets into one (see the Combining datasets section).

The command-line options for Snakemake can be set in a YAML file that specifies the execution profile. The default profile provided contains the settings needed by the workflow (Supplementary File 5).

After editing the configuration file and execution profile, the user only needs to run the following to execute WeavePop:

conda activate snakemake

snakemake --profile config/default

### Modules

The 9 modules of the WeavePop workflow, including the tasks they accomplish and the tools employed, are described below.

#### 1. Processing of reference genomes

This module standardizes the format of the reference genome annotation GFF files, homogenizes the nomenclature of feature IDs, and adds intron and intergenic features to the annotation. These steps are implemented using AGAT (v1.4.0; Dainat 2022) and Python. Optionally, RepeatModeler and RepeatMasker can be used to identify repetitive sequences in the reference genomes.

For this module, the inputs are a FASTA file and a GFF annotation file for each reference genome. Alternatively, if homogeneous annotation for all references is desired or there are no annotations available for all references, the user can provide a single GFF file, which will be used to annotate the rest of the references via Liftoff (v1.6.3; Shumate and Salzberg 2021). Doing so facilitates the comparison of variation in genic regions and other annotated features even when samples are aligned to different references.

#### 2. Read mapping, variant calling, and assembly

Snippy (v4.6.0; Seemann 2020), a haploid variant calling pipeline, performs the central analysis of the WeavePop workflow, using the paired-end short reads of each sample and the corresponding reference genome. Snippy uses BWA (Li and Durbin 2009) to align the reads to the reference andfreebayes (Garrison and Marth 2012) to call small variants. It also generates a reference-based assembly, which is a version of the reference genome with the called variants instantiated.

#### 3. Depth and quality

To analyze the quality of the read alignments of each sample, several metrics of read depth, mapping quality (MAPQ), and coverage are obtained in this module.SAMtools (v1.20; Li et al. 2009) is used to filter the BAM file with a user-defined threshold (min_mapq) for the MAPQ of the aligned reads. The stats command fromSAMtools is then used to get the read depth distribution of each chromosome from the raw and filtered BAM files. From the distributions, the mean and median depth of each chromosome and the genome-wide mean and median are obtained.SAMtools stats is also used to obtain, from the raw BAM file, the percentage of unmapped, mapped, and properly paired reads, along with the percentage of reads with low, intermediate, and high MAPQ, delimited by user-defined thresholds. The coverage command ofSAMtools is used to get the coverage of the raw and filtered reads. These metrics are used to flag samples according to user-defined thresholds: minimum percentages of high-quality alignments and mapped reads, coverage, and genome-wide median depth. The flagged samples can be excluded from all further steps.

Finally, to estimate the mean read depth of windows of a fixed size (specified by the user) along each chromosome, to be used in modules 6 and 7, Mosdepth (v0.3.8; Pedersen and Quinlan 2018) is used on the high-quality reads.

#### 4. Annotation of SNP effects

This module predicts the coding and modifier effects of small variants. The VCF files of the samples that were aligned to a reference are compiled, using the BCFtoolsisec command (v1.20; Danecek et al. 2021), to obtain a VCF of all the variants in that group of samples, along with a presence/absence matrix of the variants in each sample. The new VCF file is then annotated with SnpEff (v5.2; Cingolani et al. 2012) using the annotation of the corresponding reference. The annotations in the VCF are then transferred to multiple tables. An additional table is created with each variant classified by the highest impact among its predicted effects, and whether it is private or not.

#### 5. Annotation

Liftoff annotates each sample's reference-based assembly using the annotation of the corresponding reference. Then AGAT adds the intergenic and intronic features to the GFF files and extracts the sequences of the annotated transcripts of all genes. This produces a FASTA file per sample with all the predicted nucleotide coding sequences and another with the corresponding amino acid sequences.

#### 6. Depth and quality of genes

This module implements windowed analyses of mapped read depth and quality using the Mosdepth results from module 3, and SAMtools mpileup, along withBEDTools (v2.31.1; Quinlan and Hall 2010), to estimate the mean MAPQ of the windows. Per-window metrics of read depth and quality are intersected with the annotation to estimate the mean depth and mean MAPQ of each annotated genomic feature, including each gene and its nested features.

#### 7. CNV calling

In this module, CNVs are identified using the read depth estimates obtained with Mosdepth in module 3. The median depth of all the windows in the genome is used to normalize the mean depth of each window along the chromosomes. The normalized read depth is then smoothed using SciPy's ndimage median filter algorithm (v1.14.1; Virtanen et al. 2020) with a smoothing size specified by the user. Each window is classified as deleted, duplicated, or single copy according to a user-defined threshold based on the smoothed normalized read depth values. Additionally, BEDTools is used to calculate the fraction of each window that overlaps with repetitive sequences of the corresponding reference genome if they were identified in module 1. Consecutive windows with the same classification are fused into regions, and duplicated/deleted regions are catalogued in a table. A second table summarizing the full set of CNVs for each chromosome is also generated, with metrics such as the number of regions in the chromosome and the percentage of chromosomal length covered by each CNV type.

In contrast to other published CNV-calling algorithms (Suvakov et al. 2021; Schikora-Tamarit and Gabaldón 2022), WeavePop does not implement depth corrections for GC content, mappability, or telomere distance, but instead relies on user-defined normalized depth thresholds. The relative simplicity of WeavePop's CNV-calling algorithm makes it significantly faster than the other tools we compared it to, and despite the simplicity, it performs similarly to such tools. On average, for CNV calls in 500-bp windows, we find 97.3% agreement between WeavePop and PerSVade (v1.02.6), and 96.8% agreement with CNVpytor (v1.3.2), compared to 97.3% agreement between PerSVade and CNVpytor. Where the 3 algorithms disagree is in regions of repetitive sequences (Supplementary Methods).

#### 8. Database

The main outputs of the workflow are combined into a relational database to facilitate querying and exploration. WeavePop employs the DuckDB database system, a portable and efficient platform that supports a rich SQL dialect and provides APIs for many commonly used programming languages such as R and Python. The DuckDB database that WeavePop creates is a single-file database containing the key tables generated by modules 4, 6, and 7, the sequences of the annotated features, the annotation tables of the reference genomes, and the user-provided metadata table. The schema of the database can be found in Supplementary Fig. 2.

**Fig. 2.**
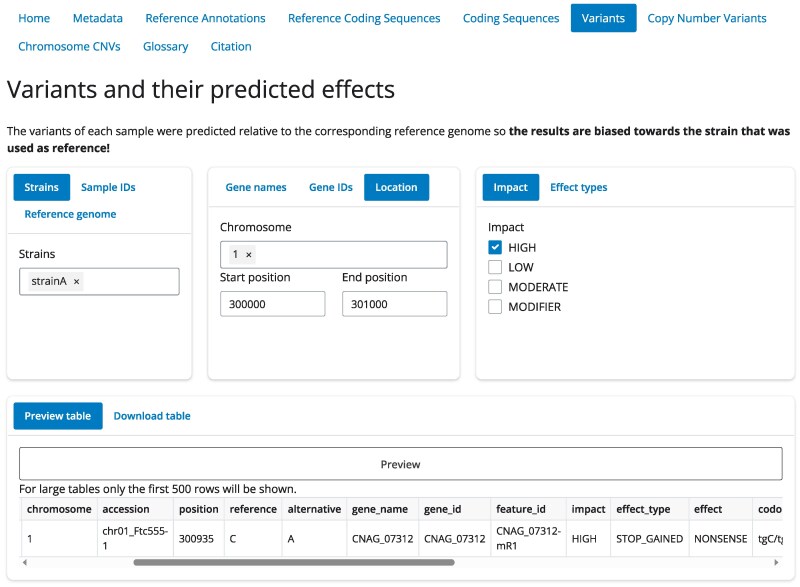
Example of WeavePop-Shiny. It shows the Variants tab with an example of a combination of filters to obtain the high-impact variants in a specific range of coordinates of one strain.

The WeavePop database can be used to extract results of interest in a simpler and more powerful manner than from the original tables and files alone. Since this is a relational SQL database, multiple pieces of information from different tables can be combined using SQL queries to obtain specific outputs of interest, such as all the genes in a certain chromosomal region that have variants with high-impact effects. Using the database is further simplified using the provided tool, WeavePop-Shiny, a Shiny for Python application. WeavePop-Shiny provides a graphical web interface that helps users unfamiliar with SQL to filter and extract results from the database using predefined functions that generate output tables (Fig. 2). For integration with other command-line tools, we also provide a command-line interface, WeavePop-CLI, that implements the same functions as WeavePop-Shiny while also allowing for arbitrary SQL queries. Examples of the use of WeavePop-CLI are given in Supplementary File 6.

#### 9. Plotting

Results from modules 1, 3, 4, 6, and 7 are used to create plots, using ggplot2 (v3.4.1; Wickham 2016), that show metrics about the mapping quality, depth, CNV, and variants, as well as the chromosomal distribution of variants, read depth, and MAPQ for each sample. An optional list of gene IDs provided by the user can be used to obtain the coordinates of the features from the reference genome to include user-defined genetic features in these last 2 plots (e.g. centromere flanking genes, mating type locus, and individual genes of interest).

### Output

In addition to the DuckDB database, all intermediate and final outputs produced by WeavePop are organized into a hierarchical directory structure. This directory structure has been designed to allow for flexibility in downstream analyses as well as exploration of alternate tool chains. The directory structure was also designed to minimize additional computation required if a subset of samples is changed or a set of analyses is re-run. A full description of the output files is given in Supplementary File 7.

### Combining datasets

Recognizing that population genomic resources grow over time, we have implemented facilities for combining the outputs of multiple WeavePop runs. This allows for simple integration of samples from different sequencing projects or data from multiple labs, avoiding redundant computation and storage as more data is obtained. To achieve this, we created an alternative workflow called “join_datasets” that takes as input 1 directory with the results of the main “analysis” workflow per dataset. It joins the tables that go into the database, and it repeats the SnpEff module using all the samples, giving as output a database with the samples from all input datasets.

## Results and discussion

To demonstrate the use of WeavePop, we re-analyzed the genomes of 1,016 isolates of the basidiomycete fungus *C. neoformans* (Desjardins et al. 2017; Ashton et al. 2019). *C. neoformans* is an opportunistic pathogen and is considered a critical priority pathogen by the World Health Organization (WHO 2022). Here, we demonstrate an analysis of chromosomal structural variation, facilitated by the CNV calling pipeline of WeavePop. Aneuploidy and large-scale copy number variation have been shown to contribute to traits such as resistance to antifungal drugs in *Cryptococcus* and other fungal pathogens (Morrow and Fraser 2013). The brief analysis here focuses on differences in aneuploidy and CNV between the major lineages of *C. neoformans* and between environmental and clinical strains. A more detailed analysis of *C. neoformans* structural variation will be presented elsewhere.

### *C. neoformans* aneuploidy and large-scale copy number variation

To identify aneuploidy and large-scale copy number variation in *C. neoformans,* we combined and re-analyzed genome data from 2 earlier studies on the global genomic diversity of this species. The study by Desjardins et al. (2017) reported genome data for 387 *C. neoformans* strains, representing each of the 4 major lineages (VNI, VNII, VNBI, and VNBII), and including both environmental and clinical isolates. A later study by Ashton et al. (2019) described a sample of 678 isolates of the VNI lineage collected from various countries in Asia. We used the dataset joining facilities of WeavePop to conduct a joint analysis of the strains from these 2 data sources. After removing 49 samples due to low quality or uncertain ploidy, we analyzed a final dataset of 1,016 isolates (Supplementary Methods, Files 8 and 9, and Tables 1 to 3).

**Fig. 3.**
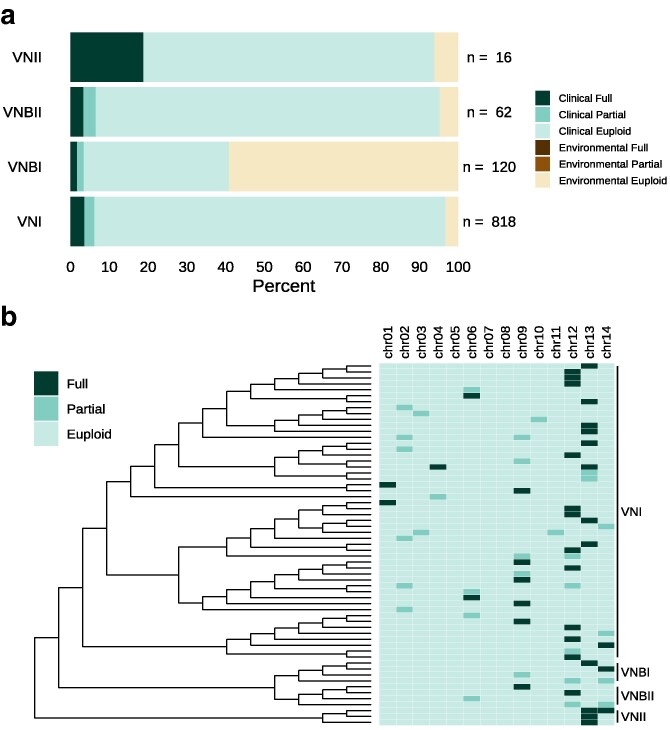
Copy number variation in *C. neoformans*. a) Percentage of samples per lineage and source of isolation with at least 1 fully duplicated chromosome (full: ≥80% of chromosome length covered by called duplications; in VNI *n* = 29, in VNII *n* = 3, and VNBI and VNBII *n* = 2), at least 1 partially duplicated chromosome (partial: 20% to 80%; in VNI *n* = 21, in VNBI and VNBII *n* = 2), or no duplication (euploid: 0% to 20%). The bars represent the percentage of samples in each category for each lineage, and the numbers to the right of the bars indicate the total number of samples analyzed per lineage. b) Reduced phylogeny of the samples with chromosomes fully or partially duplicated. The right section has 1 column per chromosome with cells colored by category of duplication.

We used the results of the WeavePop CNV-calling module to distinguish aneuploid and partially duplicated chromosomes (Supplementary Methods and Table 4) in this dataset. None of the 1,016 isolates contains deleted chromosomes, but 36 strains (3.5%), all of clinical origin, have at least 1 fully duplicated chromosome (Fig. 3a; Supplementary Fig. 3). When considering partially duplicated chromosomes (20% to 80% of their length present in 2 or more copies), we identified an additional 25 strains, all clinical isolates as well (Fig. 3a). In sum, nearly 6% of *C. neoformans* strains have large-scale chromosomal duplications. The chromosomes that we found to be most frequently fully duplicated were chromosomes 12 and 13, which were aneuploid in 12 strains each (Fig. 3b; Supplementary Fig. 4). Our finding is consistent with several other studies that identified chromosomes 12 and 13 among the most frequently duplicated chromosomes (Rhodes et al. 2017; Stott et al. 2024). Duplicated copies of chromosomes 1, 4, and 6 were only found in isolates of lineage VNI; chromosome 9 and 12 duplications were only found in samples of lineages VNI and VNBII, and chromosomes 13 and 14 in samples of lineages VNI, VNBI, and VNII (Fig. 3b). Within the lineage VNI, for which we have the most samples (*n* = 818), 29 strains had fully duplicated chromosomes (3.5%). Chromosomes 4 and 14 were duplicated in 1 isolate each; chromosomes 1 and 6 in 2, chromosome 9 in 5, chromosome 13 in 8, and chromosome 12 in 11 (Fig. 3b).

An important caveat for all large-scale analyses of copy number variation in microbial organisms is the possibility that duplicated chromosomes may be lost with passaging prior to genome sequencing. For example, in *C. neoformans* chromosome 1, aneuploidy has been shown to be selected for under exposure to the antifungal drug fluconazole (Sionov et al. 2010). However, chromosome 1 aneuploidy tends to be highly deleterious in the absence of azole drugs and is typically lost after a modest number of passages on nonselective media. The number of passages that the isolates analyzed here underwent prior to genome sequencing is unknown, and hence we cannot rule out the possibility that postcollection selection against aneuploidy biases the chromosomal patterns we detected.

### Related tools

There is an abundance of computational programs that identify genomic variants from short-read sequences and reference genomes. Some are focused on small variants (single and multiple nucleotide polymorphisms and indels), and others are focused on large variants (CNVs and/or complex structural variants). However, to our knowledge, only a few workflows include both kinds of variants.

Similar to WeavePop, PerSVade (Schikora-Tamarit and Gabaldón 2022) identifies both small- and large-scale genomic variation, including variant calling with effect prediction, estimated read depth of genes, and large CNVs. PerSVade identifies complex structural variants with personalized parameters, making it a more suitable tool for detailed analysis of individual samples. A key difference that gives WeavePop a special purpose is the fact that it can efficiently analyze large numbers of samples and give population-scale results. A second workflow that also detects small and large genomic variation is nf-core/sarek (Hanssen et al. 2024). This tool includes many software options for each step and is designed to work on multiple samples, but it is designed primarily for biomedical research on humans and mice.

In comparison to the available tools, WeavePop has a unique advantage in that it generates a user-friendly and shareable database that facilitates a quick exploration, without the need to manipulate tables or extract information from files of specific formats.

### Conclusion

WeavePop is a reproducible, readable, customizable, and scalable workflow for genomic analysis of haploid eukaryotic populations. It uses field-standard programs to detect small variants and annotate their effect, identify CNVs, and provide reference-based assemblies, annotation, and sequences of multiple samples. It generates a database to explore variation in a population, which can be used as a shared resource for research communities. A future version of WeavePop is intended to include the discovery of complex structural variants and the possibility of analyzing diploid genomes.

## Supplementary Material

jkag039_Supplementary_Data

## Data Availability

WeavePop is available at GitHub https://github.com/magwenelab/WeavePop, which includes instructions, examples, and recommendations for users in the repository's Wiki https://github.com/magwenelab/WeavePop/wiki. The repository describing the analysis of the *C. neoformans* strain collection using WeavePop is available at https://github.com/magwenelab/WeavePop_Cneoformans and https://doi.org/10.5281/zenodo.18600877. The supplementary files and tables are included in the GitHub repositories mentioned and listed in the Supplementary material. The sequencing data and reference genomes used were obtained from NCBI and FungiDB, and their accession numbers are listed in the Supplementary Methods. The authors affirm that all data necessary for confirming the conclusions of the article are present within the article, figures, tables, and the linked materials described above. Supplementary material available at *G3* online.

## References

[jkag039-B1] Ashton PM et al 2019. Three phylogenetic groups have driven the recent population expansion of *Cryptococcus neoformans*. Nat Commun. 10:2035. 10.1038/s41467-019-10092-5. 31048698 PMC6497710

[jkag039-B2] Bao W, Kojima KK, Kohany O. 2015. Repbase update, a database of repetitive elements in eukaryotic genomes. Mob DNA. 6:11. 10.1186/s13100-015-0041-9. 26045719 PMC4455052

[jkag039-B3] Brown GD et al 2024. The pathobiology of human fungal infections. Nat Rev Microbiol. 22:687–704. 10.1038/s41579-024-01062-w. 38918447

[jkag039-B4] Cingolani P et al 2012. A program for annotating and predicting the effects of single nucleotide polymorphisms, SnpEff: SNPs in the genome of Drosophila melanogaster strain w^1118^ ; iso-2; iso-3. Fly (Austin). 6:80–92. 10.4161/fly.19695. 22728672 PMC3679285

[jkag039-B5] Conda Contributors . 2025. Conda: A system-level, binary package and environment manager running on all major operating systems and platforms. https://docs.conda.io/en/latest/

[jkag039-B6] Dainat J . 2022. AGAT: Another Gff Analysis Toolkit to handle annotations in any GTF/GFF format. 10.5281/zenodo.3552717

[jkag039-B7] Danecek P et al 2021. Twelve years of SAMtools and BCFtools. GigaScience. 10:giab008. 10.1093/gigascience/giab008. 33590861 PMC7931819

[jkag039-B8] Desjardins CA et al 2017. Population genomics and the evolution of virulence in the fungal pathogen *Cryptococcus neoformans*. Genome Res. 27:1207–1219. 10.1101/gr.218727.116. 28611159 PMC5495072

[jkag039-B9] Flynn JM et al 2020. RepeatModeler2 for automated genomic discovery of transposable element families. Proc Natl Acad Sci U S A. 117:9451–9457. 10.1073/pnas.1921046117. 32300014 PMC7196820

[jkag039-B10] Garrison E, Marth G. 2012. Haplotype-based variant detection from short-read sequencing [Preprint]. arXiv 3907. [accessed 2026 Feb 3]. 10.48550/arXiv.1207.3907

[jkag039-B11] Hanssen F et al 2024. Scalable and efficient DNA sequencing analysis on different compute infrastructures aiding variant discovery. NAR Genomics Bioinforma. 6:lqae031. 10.1093/nargab/lqae031. PMC11044436 38666213

[jkag039-B12] Li H et al 2009. The sequence alignment/map format and SAMtools. Bioinformatics. 25:2078–2079. 10.1093/bioinformatics/btp352. 19505943 PMC2723002

[jkag039-B13] Li H, Durbin R. 2009. Fast and accurate short read alignment with Burrows–Wheeler transform. Bioinformatics. 25:1754–1760. 10.1093/bioinformatics/btp324. 19451168 PMC2705234

[jkag039-B14] Mölder F et al 2021. Sustainable data analysis with Snakemake. F1000Res. 10:33. https://f1000research.com/articles/10-33/v3 10.12688/f1000research.29032.3. 34035898 PMC8114187

[jkag039-B15] Morrow CA, Fraser JA. 2013. Ploidy variation as an adaptive mechanism in human pathogenic fungi. Semin Cell Dev Biol. 24:339–346. 10.1016/j.semcdb.2013.01.008. 23380396

[jkag039-B16] Pedersen BS, Quinlan AR. 2018. Mosdepth: quick coverage calculation for genomes and exomes. Bioinformatics. 34:867–868. 10.1093/bioinformatics/btx699. 29096012 PMC6030888

[jkag039-B17] Posit Software . 2022. Shiny for Python. https://shiny.posit.co/py/

[jkag039-B18] Quinlan AR, Hall IM. 2010. BEDTools: a flexible suite of utilities for comparing genomic features. Bioinformatics. 26:841–842. 10.1093/bioinformatics/btq033. 20110278 PMC2832824

[jkag039-B19] Raasveldt M, Mühleisen H. 2019. DuckDB: an embeddable analytical database. Proceedings of the 2019 International Conference on Management of Data. Amsterdam, Netherlands. ACM. p. 1981–1984. 10.1145/3299869.3320212.

[jkag039-B20] Rhodes J et al 2017. A population genomics approach to assessing the genetic basis of within-host microevolution underlying recurrent cryptococcal meningitis infection. G3 (Bethesda). 7:1165–1176. 10.1534/g3.116.037499. 28188180 PMC5386865

[jkag039-B21] Schikora-Tamarit MÀ, Gabaldón T. 2022. PerSVade: personalized structural variant detection in any species of interest. Genome Biol. 23:175. 10.1186/s13059-022-02737-4. 35974382 PMC9380391

[jkag039-B22] Seemann T . 2020. Snippy. https://github.com/tseemann/snippy.

[jkag039-B23] Shumate A, Salzberg SL. 2021. Liftoff: accurate mapping of gene annotations. Bioinformatics. 37:1639–1643. 10.1093/bioinformatics/btaa1016. 33320174 PMC8289374

[jkag039-B24] Sionov E, Lee H, Chang YC, Kwon-Chung KJ. 2010. *Cryptococcus neoformans* overcomes stress of azole drugs by formation of disomy in specific multiple chromosomes. PLoS Pathog. 6:e1000848. 10.1371/journal.ppat.1000848. 20368972 PMC2848560

[jkag039-B25] Smit A . 2021. RepeatMasker. https://www.repeatmasker.org/.

[jkag039-B26] Stott KE et al 2024. Integration of genomic and pharmacokinetic data to predict clinical outcomes in HIV-associated cryptococcal meningitis. mBio. 15:e0159224. 10.1128/mbio.01592-24. 39189739 PMC11481554

[jkag039-B27] Suvakov M, Panda A, Diesh C, Holmes I, Abyzov A. 2021. CNVpytor: a tool for copy number variation detection and analysis from read depth and allele imbalance in whole-genome sequencing. GigaScience. 10:giab074. 10.1093/gigascience/giab074. 34817058 PMC8612020

[jkag039-B28] Virtanen P et al 2020. Scipy 1.0: fundamental algorithms for scientific computing in Python. Nat Methods. 17:261–272. 10.1038/s41592-019-0686-2. 32015543 PMC7056644

[jkag039-B29] WHO . 2022. WHO fungal priority pathogens list to guide research, development and public health action. https://www.who.int/publications/i/item/9789240060241.

[jkag039-B30] Wickham H . 2016. Ggplot2: elegant graphics for data analysis. 2nd ed. Springer Verlag. https://ggplot2.tidyverse.org.

