# Supplementary material for: WeavePop: a bioinformatics workflow to explore and analyze genomic variants of eukaryotic populations
Source: G3 (Bethesda). 2026 Feb 13;16(4):jkag039. doi: 10.1093/g3journal/jkag039 (PMC13042275; doi:10.1093/g3journal/jkag039)
Supplement: jkag039_Supplementary_Data [file jkag039_supplementary_data.zip › Figure_S1_G3-2025-406398.pdf]

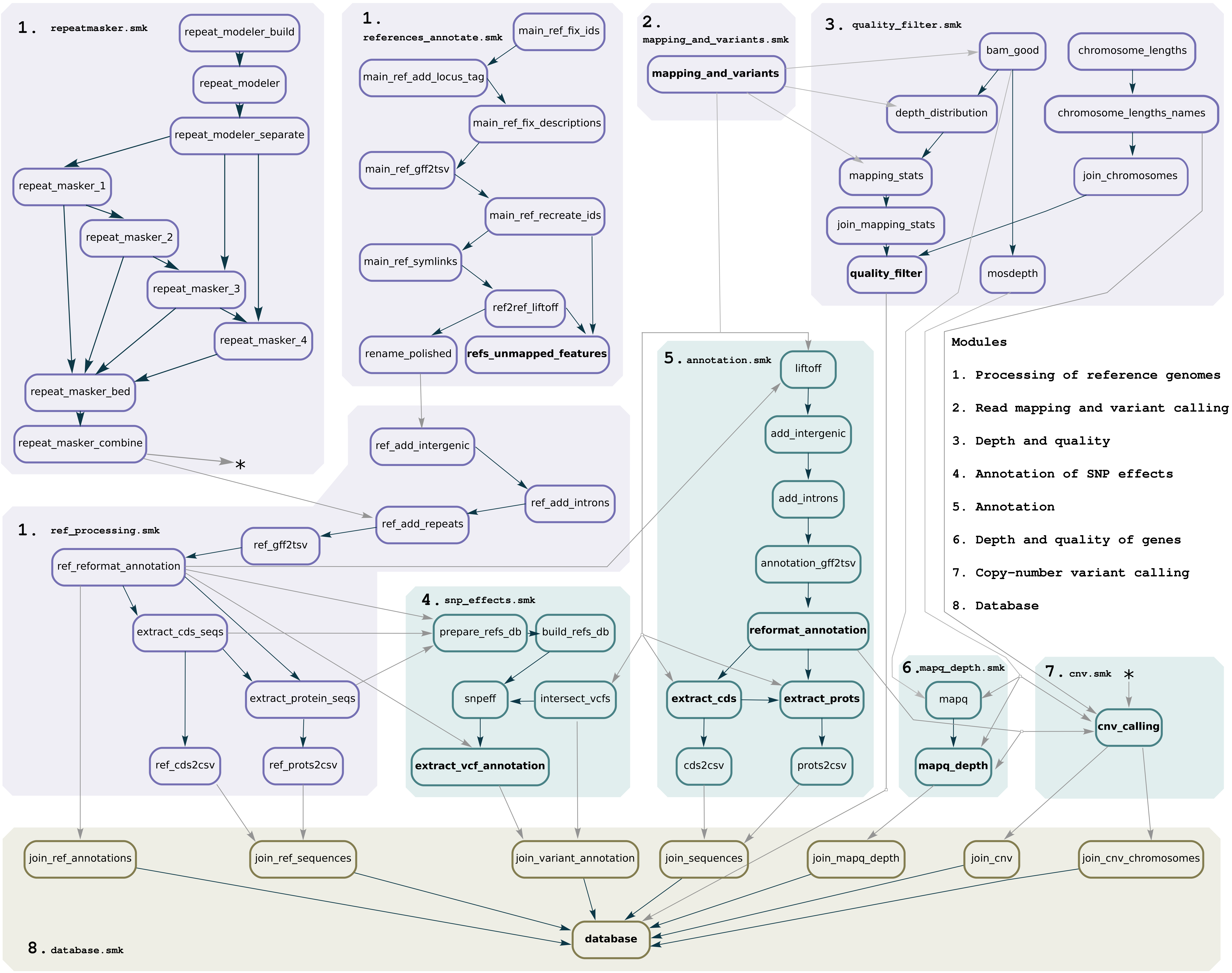
1. repeatmasker.smk
repeat_modeler_build
repeat_modeler
repeat_modeler_separate
repeat_masker_1
repeat_masker_2
repeat_masker_3
repeat_masker_4
repeat_masker_bed
repeat_masker_combine
*
1. references_annotate.smk
main_ref_fix_ids
main_ref_add_locus_tag
main_ref_fix_descriptions
main_ref_gff2tsv
main_ref_recreate_ids
main_ref_symlinks
ref2ref_liftoff
rename_polished
refs_unmapped_features
2. mapping_and_variants.smk
mapping_and_variants
3. quality_filter.smk
bam_good
chromosome_lengths
depth_distribution
chromosome_lengths_names
mapping_stats
join_chromosomes
join_mapping_stats
quality_filter
mosdepth
ref_add_intergenic
ref_add_introns
ref_add_repeats
1. ref_processing.smk
ref_gff2tsv
ref_reformat_annotation
extract_cds_seqs
extract_protein_seqs
ref_cds2csv
ref_prots2csv
4. snp_effects.smk
prepare_refs_db
build_refs_db
snpeff
intersect_vcfs
extract_vcf_annotation
5. annotation.smk
liftoff
add_intergenic
add_introns
annotation_gff2tsv
reformat_annotation
extract_cds
extract_prots
cds2csv
prots2csv
6. mapq_depth.smk
mapq
mapq_depth
7. cnv.smk
*
cnv_calling
Modules
1. Processing of reference genomes
2. Read mapping and variant calling
3. Depth and quality
4. Annotation of SNP effects
5. Annotation
6. Depth and quality of genes
7. Copy-number variant calling
8. Database
join_ref_annotations
join_ref_sequences
join_variant_annotation
join_sequences
join_mapq_depth
join_cnv
join_cnv_chromosomes
database
8. database.smk

Figure S1. Directed Acyclic Graph (DAG) of jobs in the WeavePop workflow, with the identification of repetitive sequences, the annotation of reference genomes, and the production of the database activated. The trivial rules ref_fasta_symlinks and agat_config were omitted for simplicity. The rules are located in boxes corresponding to the files where they are defined.
