# Supplementary material for: WeavePop: a bioinformatics workflow to explore and analyze genomic variants of eukaryotic populations
Source: G3 (Bethesda). 2026 Feb 13;16(4):jkag039. doi: 10.1093/g3journal/jkag039 (PMC13042275; doi:10.1093/g3journal/jkag039)
Supplement: jkag039_Supplementary_Data [file jkag039_supplementary_data.zip › Figure_S2_G3-2025-406398.pdf]

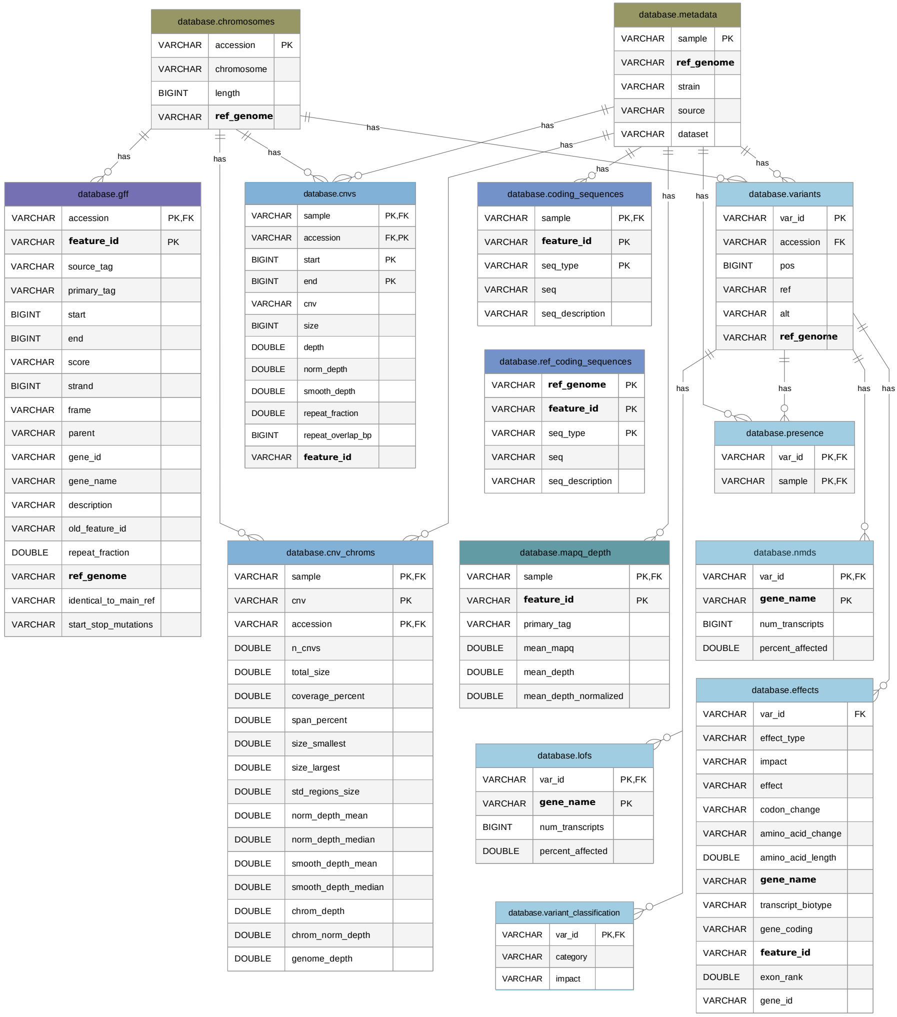
database.chromosomes
VARCHAR accession PK
VARCHAR chromosome
BIGINT length
VARCHAR ref_genome
database.metadata
VARCHAR sample PK
VARCHAR ref_genome
VARCHAR strain
VARCHAR source
VARCHAR dataset
has
database.gff
VARCHAR accession PK,FK
VARCHAR feature_id PK
VARCHAR source_tag
VARCHAR primary_tag
BIGINT start
BIGINT end
VARCHAR score
BIGINT strand
VARCHAR frame
VARCHAR parent
VARCHAR gene_id
VARCHAR gene_name
VARCHAR description
VARCHAR old_feature_id
DOUBLE repeat_fraction
VARCHAR ref_genome
VARCHAR identical_to_main_ref
VARCHAR start_stop_mutations
database.cnvs
VARCHAR sample PK,FK
VARCHAR accession FK,PK
BIGINT start PK
BIGINT end PK
VARCHAR cnv
BIGINT size
DOUBLE depth
DOUBLE norm_depth
DOUBLE smooth_depth
DOUBLE repeat_fraction
BIGINT repeat_overlap_bp
VARCHAR feature_id
database.coding_sequences
VARCHAR sample PK,FK
VARCHAR feature_id PK
VARCHAR seq_type PK
VARCHAR seq
VARCHAR seq_description
database.ref_coding_sequences
VARCHAR ref_genome PK
VARCHAR feature_id PK
VARCHAR seq_type PK
VARCHAR seq
VARCHAR seq_description
database.variants
VARCHAR var_id PK
VARCHAR accession FK
BIGINT pos
VARCHAR ref
VARCHAR alt
VARCHAR ref_genome
database.presence
VARCHAR var_id PK,FK
VARCHAR sample PK,FK
database.cnv_chroms
VARCHAR sample PK,FK
VARCHAR cnv PK
VARCHAR accession PK,FK
DOUBLE n_cnvs
DOUBLE total_size
DOUBLE coverage_percent
DOUBLE span_percent
DOUBLE size_smallest
DOUBLE size_largest
DOUBLE std_regions_size
DOUBLE norm_depth_mean
DOUBLE norm_depth_median
DOUBLE smooth_depth_mean
DOUBLE smooth_depth_median
DOUBLE chrom_depth
DOUBLE chrom_norm_depth
DOUBLE genome_depth
database.mapq_depth
VARCHAR sample PK,FK
VARCHAR feature_id PK
VARCHAR primary_tag
DOUBLE mean_mapq
DOUBLE mean_depth
DOUBLE mean_depth_normalized
database.nmds
VARCHAR var_id PK,FK
VARCHAR gene_name PK
BIGINT num_transcripts
DOUBLE percent_affected
database.effects
VARCHAR var_id FK
VARCHAR effect_type
VARCHAR impact
VARCHAR effect
VARCHAR codon_change
VARCHAR amino_acid_change
DOUBLE amino_acid_length
VARCHAR gene_name
VARCHAR transcript_biotype
VARCHAR gene_coding
VARCHAR feature_id
DOUBLE exon_rank
VARCHAR gene_id
database.lofs
VARCHAR var_id PK,FK
VARCHAR gene_name PK
BIGINT num_transcripts
DOUBLE percent_affected
database.variant_classification
VARCHAR var_id PK,FK
VARCHAR category
VARCHAR impact

Figure S2. Entity relationship diagram of the database generated by WeavePop. The columns that identify each observation on their own or in combination are defined as primary keys (PK), and the columns that refer to a primary key of another table are defined as foreign keys (FK). Other columns that are common to many tables but are not a unique primary key in any of them are in bold. The tables that are created by the same module have the same color.
