# Supplementary material for: WeavePop: a bioinformatics workflow to explore and analyze genomic variants of eukaryotic populations
Source: G3 (Bethesda). 2026 Feb 13;16(4):jkag039. doi: 10.1093/g3journal/jkag039 (PMC13042275; doi:10.1093/g3journal/jkag039)
Supplement: jkag039_Supplementary_Data [file jkag039_supplementary_data.zip › Figure_S3_G3-2025-406398.pdf]

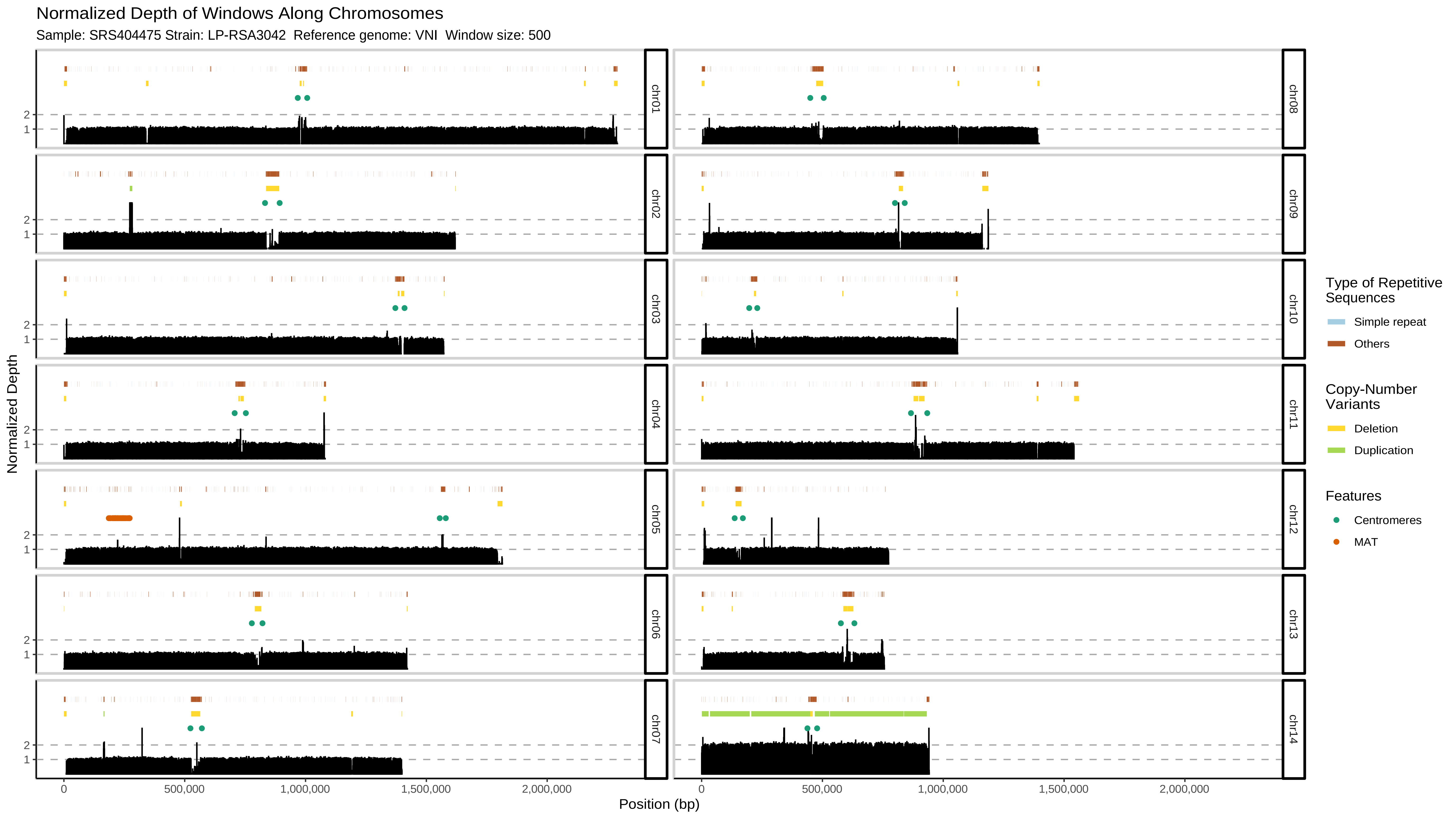
Normalized Depth of Windows Along Chromosomes
Sample: SRS404475 Strain: LP-RSA3042 Reference genome: VNI Window size: 500
Normalized Depth
Position (bp)
chr01
chr02
chr03
chr04
chr05
chr06
chr07
chr08
chr09
chr10
chr11
chr12
chr13
chr14
0
500,000
1,000,000
1,500,000
2,000,000
1
2
Type of Repetitive Sequences
Simple repeat
Others
Copy-Number Variants
Deletion
Duplication
Features
Centromeres
MAT

Figure S3. Plot generated by WeavePop showing the normalized read depth of 500 bp windows along all chromosomes of the strain LP-RSA3042. Above the bars showing the read depth, the top track shows segments covered by repetitive sequences in the corresponding reference, the second one shows the identified CNV regions, and the third one shows the location of the genes flanking the centromeres and the mating-type locus. Chromosome 14 is fully duplicated.
