# Supplementary material for: WeavePop: a bioinformatics workflow to explore and analyze genomic variants of eukaryotic populations
Source: G3 (Bethesda). 2026 Feb 13;16(4):jkag039. doi: 10.1093/g3journal/jkag039 (PMC13042275; doi:10.1093/g3journal/jkag039)
Supplement: jkag039_Supplementary_Data [file jkag039_supplementary_data.zip › Figure_S4_G3-2025-406398.pdf]

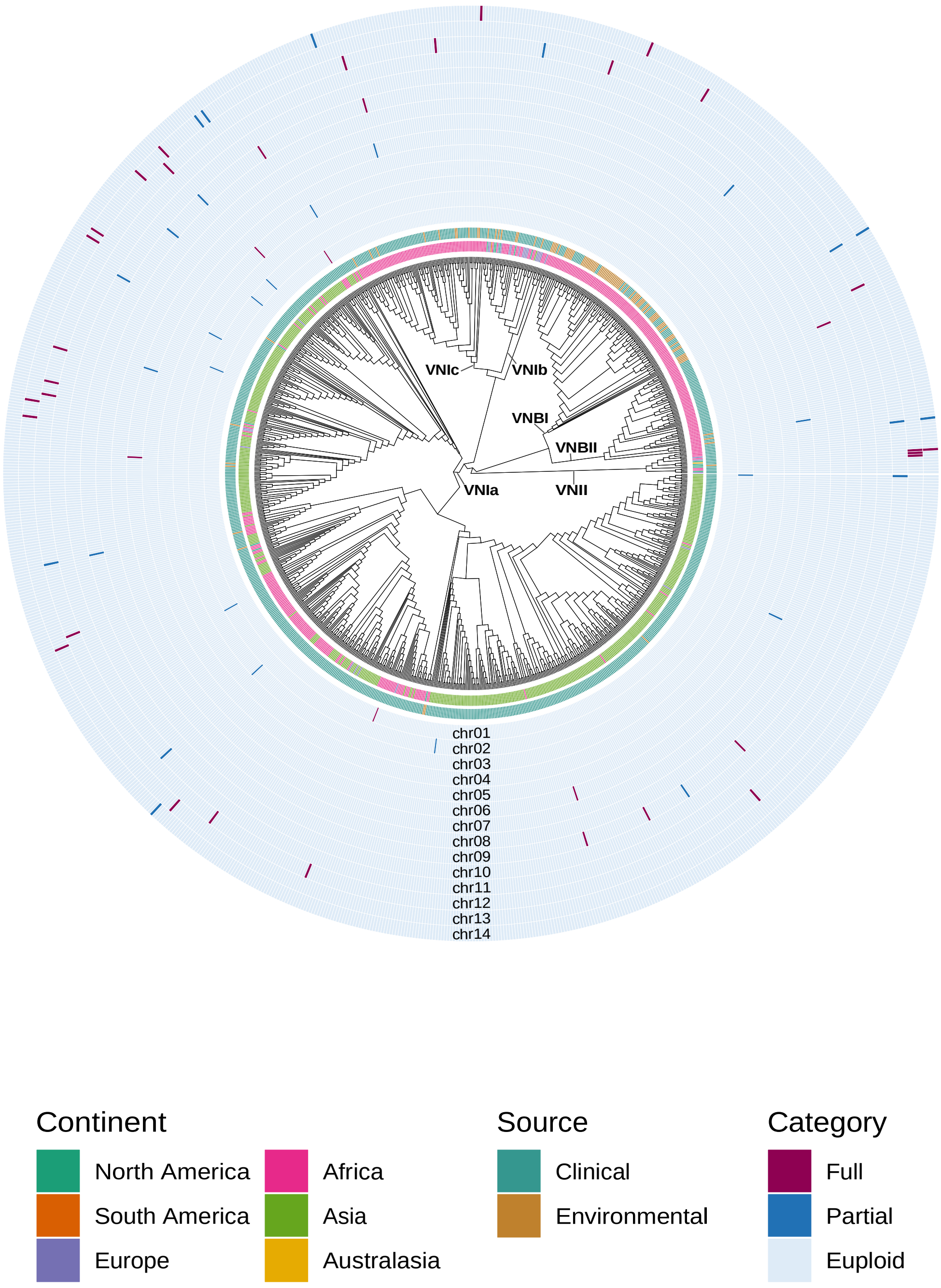
VNIc
VNIb
VNBI
VNBII
VNIa
VNII
chr01
chr02
chr03
chr04
chr05
chr06
chr07
chr08
chr09
chr10
chr11
chr12
chr13
chr14
Continent
North America
South America
Europe
Africa
Asia
Australasia
Source
Clinical
Environmental
Category
Full
Partial
Euploid

Figure S4. Phylogeny of C. neoformans with fully and partially duplicated chromosomes in 1,016 analyzed strains and the reference strain H99. The outer section has one ring per chromosome (in ascending order from the center) with cells colored by category of duplication (Full: >=80% of chromosome length covered by called duplications; Partial: 20-80%; Euploid: 0-20%). The cladogram was constructed by replacing the VNI clade in the phylogeny of Desjardins et al. (2017) with the phylogeny of Ashton et al. (2019) (which includes the VNI Desjardins samples).
