# Supplementary material for: WeavePop: a bioinformatics workflow to explore and analyze genomic variants of eukaryotic populations
Source: G3 (Bethesda). 2026 Feb 13;16(4):jkag039. doi: 10.1093/g3journal/jkag039 (PMC13042275; doi:10.1093/g3journal/jkag039)
Supplement: jkag039_Supplementary_Data [file jkag039_supplementary_data.zip › Supplementary_Data_G3-2025-406398.docx]

### Supplementary Data

File S1: Input metadata table example:

<https://github.com/magwenelab/WeavePop/blob/main/test/config/metadata.csv>

File S2: Input chromosomes table example:

<https://github.com/magwenelab/WeavePop/blob/main/test/config/chromosomes.csv>

File S3: Input loci table example:

<https://github.com/magwenelab/WeavePop/blob/main/test/config/loci.csv>

File S4: Configuration file:

<https://github.com/magwenelab/WeavePop/blob/main/config/config.yaml>

File S5: Execution profile (YAML with Snakemake command-line options):

<https://github.com/magwenelab/WeavePop/blob/main/config/default/config.yaml>

File S6: WeavePop-CLI examples:

<https://github.com/magwenelab/WeavePop/wiki/WeavePop%E2%80%90CLI>

File S7: Wiki with description of the output:

<https://github.com/magwenelab/WeavePop/wiki/Output>

File S8: Configuration file used to analyze the Ashton dataset:

<https://github.com/magwenelab/WeavePop_Cneoformans/blob/main/Crypto_Ashton/config/config.yaml>

File S9: Configuration file used to analyze the Desjardins dataset:

<https://github.com/magwenelab/WeavePop_Cneoformans/blob/main/Crypto_Desjardins/config/config.yaml>

Table S1: Metadata table of all analyzed samples:

<https://github.com/magwenelab/WeavePop_Cneoformans/blob/main/analyses/data/processed/metadata_all.csv>

Table S2: Summary metrics per lineage:

<https://github.com/magwenelab/WeavePop_Cneoformans/blob/main/analyses/results/tables/per_lineage_summary_stats.tsv>

Table S3: Table with correspondence between accessions and chromosome names:

<https://github.com/magwenelab/WeavePop_Cneoformans/blob/main/Crypto_Desjardins/config/chromosomes.csv>

Table S4: Table with the CNV metrics per chromosome of all analyzed samples and their aneuploidy category: <https://github.com/magwenelab/WeavePop_Cneoformans/blob/main/analyses/results/tables/chromosome_cnv_categories.tsv>
