# Supplementary material for: WeavePop: a bioinformatics workflow to explore and analyze genomic variants of eukaryotic populations
Source: G3 (Bethesda). 2026 Feb 13;16(4):jkag039. doi: 10.1093/g3journal/jkag039 (PMC13042275; doi:10.1093/g3journal/jkag039)
Supplement: jkag039_Supplementary_Data [file jkag039_supplementary_data.zip › supplementary_methods.docx]

## Supplementary Methods

### Analysis of *Cryptococcus neoformans* datasets

#### Analysis of the dataset

The published datasets of short sequencing reads from Desjardins et al. [(2017)](https://www.zotero.org/google-docs/?pJCdAL)(387 samples from BioProject PRJNA382844), and Ashton et al. [(2019)](https://www.zotero.org/google-docs/?XF0Jmt) (884 samples from BioProjects PRJEB27222 and PRJEB5282) (see Table S1) were obtained from NCBI’s Sequence Read Archive. EntrezDirect [(v16.2; Kans 2013)](https://www.zotero.org/google-docs/?kKunIm) was used to get the run and sample IDs, and the SRA Toolkit [(v3.0.10; NCBI 2023)](https://www.zotero.org/google-docs/?EauGXr) was used to download the FASTQ files. One sample from the Ashton dataset was not available for download, and 21 samples did not belong to the *C. neoformans* VNI lineage and were excluded from all analyses. The samples with multiple runs were concatenated into a single file. The reads were cleaned with FastP [(Chen et al. 2018)](https://www.zotero.org/google-docs/?cpRVyu), with default parameters and the dedup option.

The reference genomes used were FungiDB release 65 (strain H99, lineage VNI), GCA_022832995.1 (strain VNII, lineage VNII), PRJNA1081442 (strain Bt22, lineage VNBI), and GCA_023650575.1 (strain Bt89, lineage VNBII). To ensure consistent naming, the reference genomes of lineages VNII, VNBI, and VNBII were aligned to the H99 genome using MashMap [(Jain et al. 2018)](https://www.zotero.org/google-docs/?NISCbK) to establish approximate chromosomal homology between them. See Table S3 for correspondence between the reference accessions. The VNI reference annotation (FungiDB release 65) was used to annotate the rest of the reference genomes in the workflow.

We ran WeavePop with parameters listed in the configuration files (Files S8 and S9). From the Ashton dataset, 9 samples had low mapping quality and were removed from further analysis. Additionally, 39 samples (8 Desjardins, 31 Ashton) have depth profiles that suggest a ploidy different than haploid and were also removed. The final results of each dataset (379 samples from Desjardins and 637 samples from Ashton) were combined using the join datasets workflow to leave a final dataset of 1,016 samples.

#### Detection of deletions and duplications in *C. neoformans*

To identify fully and partially deleted and duplicated chromosomes, we used the WeavePop output table, cnv_chromosomes.tsv, which contains the percentage of each chromosome covered by each type of CNV. Chromosomes that were 80% or more covered by deletions or duplications were classified as fully deleted or duplicated, respectively. Chromosomes whose CNV coverage was between 20% and 80% were classified as partially deleted or duplicated. And those with less than 20% of coverage by CNVs were deemed euploid (Table S4).

#### Phylogeny

To create an informal supertree with all the analyzed strains, we merged the trees published with the original datasets. The phylogeny by [Desjardins et al. (2017)](https://paperpile.com/c/2HQJMU/qORc) presents the topology among the four lineages: VNI, VNII, VNBI, and VNBII. The tree by [Ashton et al. (2019)](https://paperpile.com/c/2HQJMU/XDyc) has only the VNI lineage, but includes the strains published by them as well as the VNI strains in the Desjardins dataset. Thanks to this, we could completely substitute the VNI clade in the Desjardins tree with the full Ashton tree. We rooted the Desjardins tree in the middle of the branch leading to the VNII clade, and the Ashton tree in the middle of the branch leading to the clade VNIa. We removed the VNI clade from the Desjardins tree and used the rest of the tree as the backbone. The Ashton tree was attached to it as a sister clade to the VNB (VNBI and VNBII) lineage. This was done using the function tree.merger (Castiglione et al. 2022) of the R library RRphylo.

### Comparison of the CNV-calling module to other software

To compare the results of the CNV-calling module of WeavePop to other CNV-calling programs, we ran CNVpytor [(Suvakov et al. 2021)](https://www.zotero.org/google-docs/?usSUxU) and PerSVade [(Schikora-Tamarit and Gabaldón 2022)](https://www.zotero.org/google-docs/?MIzICk) on samples with a significant proportion of CNV regions (>=20% of at least one chromosome) called by WeavePop (n = 61). For both CNVpytor and PerSVade, we used the same reference genomes and BAM files used to call CNVs in WeavePop. For CNVpytor, we calculated GC content (-make_gc_file) of the reference genomes, and ran the program using read depth with a bin size of 500 bp. For PerSVade, we selected the algorithms HMMcopy and AneuFinder, and used a window size of 500 bp with a ploidy of 1. To make the results comparable across tools, we converted the resulting tables of CNV regions to windows of 500 bp using Bedtools intersect. For each sample, we compared these tables with the output of WeavePop's depth_by_windows.tsv and counted the percentage of windows where the call (duplication, deletion, or single copy) was the same between each pair of programs. We report the mean window agreement across all samples.

**Literature cited**

Ashton PM *et al.* 2019. Three phylogenetic groups have driven the recent population expansion of *Cryptococcus neoformans*. *Nat Commun.* 10(1):2035. https://doi.org/10.1038/s41467-019-10092-5

Castiglione S *et al.* 2022. Fast production of large, time-calibrated, informal supertrees with tree.merger. *Palaeontology.* 65(1):e12588. <https://doi.org/10.1111/pala.12588>

Chen S, Zhou Y, Chen Y, Gu J. 2018. fastp: an ultra-fast all-in-one FASTQ preprocessor. *Bioinformatics.* 34(17):i884–i890. https://doi.org/10.1093/bioinformatics/bty560

Desjardins CA *et al.* 2017. Population genomics and the evolution of virulence in the fungal pathogen *Cryptococcus neoformans*. *Genome Res.* 27(7):1207–1219. https://doi.org/10.1101/gr.218727.116

Jain C *et al.* 2018. A fast adaptive algorithm for computing whole-genome homology maps. *Bioinformatics.* 34(17):i748–i756. https://doi.org/10.1093/bioinformatics/bty597

Kans J. 2013. Entrez Direct: E‐utilities on the Unix Command Line. https://www.ncbi.nlm.nih.gov/books/NBK179288/

NCBI. 2023. SRA Toolkit. [accessed 2023 Dec 19] https://github.com/ncbi/sra-tools

Schikora-Tamarit MÀ, Gabaldón T. 2022. PerSVade: personalized structural variant detection in any species of interest. *Genome Biol.* 23(1):175. https://doi.org/10.1186/s13059-022-02737-4

Suvakov M *et al.* 2021. CNVpytor: a tool for copy number variation detection and analysis from read depth and allele imbalance in whole-genome sequencing. *GigaScience.* 10(11):giab074. https://doi.org/10.1093/gigascience/giab074
